# Dietary Patterns during Pregnancy Are Associated with Risk of Gestational Diabetes Mellitus

**DOI:** 10.3390/nu7115472

**Published:** 2015-11-12

**Authors:** Dayeon Shin, Kyung Won Lee, Won O. Song

**Affiliations:** Department of Food Science and Human Nutrition, Michigan State University, 469 Wilson Road, Trout FSHN Building, East Lansing, MI 48824, USA; shinda@msu.edu (D.S.); kyungwon@msu.edu (K.W.L.)

**Keywords:** dietary patterns, reduced rank regression, gestational diabetes mellitus, National Health and Nutrition Examination Survey

## Abstract

Maternal dietary patterns before and during pregnancy play important roles in the development of gestational diabetes mellitus (GDM). We aimed to identify dietary patterns during pregnancy that are associated with GDM risk in pregnant U.S. women. From a 24 h dietary recall of 253 pregnant women (16–41 years) included in the National Health and Nutrition Examination Survey (NHANES) 2003–2012, food items were aggregated into 28 food groups based on Food Patterns Equivalents Database. Three dietary patterns were identified by reduced rank regression with responses including prepregnancy body mass index (BMI), dietary fiber, and ratio of poly- and monounsaturated fatty acids to saturated fatty acid: “high refined grains, fats, oils and fruit juice”, “high nuts, seeds, fat and soybean; low milk and cheese”, and “high added sugar and organ meats; low fruits, vegetables and seafood”. GDM was diagnosed using fasting plasma glucose levels ≥5.1 mmol/L for gestation <24 weeks. Multivariable logistic regression models were used to estimate adjusted odds ratio (AOR) and 95% confidence intervals (CIs) for GDM, after controlling for maternal age, race/ethnicity, education, family poverty income ratio, marital status, prepregnancy BMI, gestational weight gain, energy intake, physical activity, and log-transformed C-reactive protein (CRP). All statistical analyses accounted for the appropriate survey design and sample weights of the NHANES. Of 249 pregnant women, 34 pregnant women (14%) had GDM. Multivariable AOR (95% CIs) of GDM for comparisons between the highest *vs.* lowest tertiles were 4.9 (1.4–17.0) for “high refined grains, fats, oils and fruit juice” pattern, 7.5 (1.8–32.3) for “high nuts, seeds, fat and soybean; low milk and cheese” pattern, and 22.3 (3.9–127.4) for “high added sugar and organ meats; low fruits, vegetables and seafood” pattern after controlling for maternal sociodemographic variables, prepregnancy BMI, gestational weight gain, energy intake and log-transformed CRP. These findings suggest that dietary patterns during pregnancy are associated with risk of GDM after controlling for potential confounders. The observed connection between a high consumption of refined grains, fat, added sugars and low intake of fruits and vegetables during pregnancy with higher odds for GDM, are consistent with general health benefits of healthy diets, but warrants further research to understand underlying pathophysiology of GDM associated with dietary behaviors during pregnancy.

## 1. Introduction

Gestational diabetes mellitus (GDM) is indicated when any degree of glucose intolerance is recognized for the first time during pregnancy, regardless of whether the condition may have predated the pregnancy or persisted after the pregnancy [1]. In the U.S., approximately 1%–14% of all pregnancies have been reported to be complicated by GDM, which accounts for more than 200,000 cases annually [2].

Several studies reported how macro- or micro-nutrient intakes during pregnancy are related to GDM risk [3,4,5,6]. In 171 nulliparous Chinese pregnant women, macronutrient intake estimated from a 24 h recall at 24–28 weeks of gestation were associated with glucose tolerance in pregnancy [3]. Chinese women with GDM had a significantly lower polyunsaturated fat intake (% total fat) compared to women without GDM (28.2% *vs.* 31.6% of total fat). Women with GDM had significantly higher saturated fat intake compared to those without GDM (46.1% *vs.* 42.1% of total fat) [3]. In a study of 504 Italian pregnant women, Bo *et al*. [4] found that every 10% increase in saturated fat (% total fat) at 24 to 28 weeks of gestation was associated with an increased risk for GDM, whereas every 10% increase of polyunsaturated fat (% total fat) was associated with 15% reduction of GDM risk. In a prospective cohort study entitled, Pregnancy, Infection, and Nutrition (PIN) of 1698 U.S. pregnant women, women with GDM consumed a lower percentage of energy from carbohydrates and a higher percentage of energy from fat in the second trimester than women with normal glucose tolerance did [5]. In another prospective cohort study of 3158 U.S. pregnant women, Qiu *et al.* [6] reported that the dietary heme iron intake in the first trimester was associated with an increased risk for GDM. The current body of literature indicates that high intake of saturated fat, *n*-3 fatty acids, and dietary heme iron is associated with increased risk for GDM, whereas polyunsaturated fat intake may be protective against GDM risk. However, the studies reviewed vary widely for time point of pregnancy and diet assessment, dietary assessment tools (24 h recalls *vs.* food frequency questionnaires (FFQs), and diagnostic criteria for GDM (75 g or 100 g oral glucose).

Analyses of overall food patterns account for any interactions or synergistic effects among individual foods or nutrients [7]. In literature on dietary patterns in pregnant populations, factors analysis or principal component analysis (“foods group-driven”) [8,9,10,11,12] were used to derive dietary patterns and related to pregnancy complications or birth outcomes. Reduced rank regression methods (“biomarker or nutrient-driven”) have been introduced to better assess the diet-disease relations compared to using factor analysis and principal component analysis [13], but the method has been underutilized among pregnant women. The reduced rank regression method has only been reported in the studies that assessed dietary patterns during pregnancy in relation to spina bifida [14] and congenital heart defect [15] in Netherlands. Dietary patterns derived using the reduced rank regression method is expected to explain the maximum variation of GDM-related maternal nutrients and biomarkers as response variables in women with GDM.

A few studies have examined the association between dietary patterns during pregnancy and the risk of GDM in U.S. representative pregnant women. The role of dietary patterns during pregnancy in relation to GDM risk is still uncertain. We hypothesized that dietary patterns during pregnancy derived from reduced rank regression are differentially associated with the risk of GDM.

## 2. Methods

### 2.1. Study Population

We used public domain data from the continuous National Health and Nutrition Examination (NHANES) 2003–2004, 2005–2006, 2007–2008, 2009–2010, and 2011–2012 for this study. Data from the NHANES 2003–2012 were combined for this study with greater statistical reliability. The NHANES is a program of studies cross-sectionally designed to assess the health and nutritional status of civilian, non-institutionalized population in the U.S. conducted by the National Center for Health Statistics (NCHS), Centers for Disease Control and Prevention (CDC). The NHANES used a stratified multistage probability sample that was based on the selection of counties, blocks, households, and finally persons within households. The NHANES survey is unique in that it combines interviews and physical examinations. The participants were interviewed for the information of age, race/ethnicity, education level, marital status, family poverty income ratio, and physical activity. Reproductive health interviews obtained information on month of gestation at the time of the survey. Pregnancy status was based on a positive urine pregnancy test. Prepregnancy weight was self-reported during the weight history questionnaire interview. A complete description of data-collection procedures and analytic guidelines has been provided elsewhere [16,17].

The 2003–2012 NHANES dataset included 761 pregnant women. Subjects were excluded if they reported unreliable dietary data, as defined by the NCHS (*n =* 24) and had missing data of gestational weeks (*n =* 105), measured height, weight and self-reported prepregnancy weight (*n =* 35), glucose and insulin levels (*n =* 310), and CRP levels (*n =* 1). Pregnant women who did not participate in the fasting subsample for glucose and insulin were excluded from the analysis (*n =* 33). Lastly, pregnant women who were already diagnosed with GDM were excluded (*n =* 4). The final analytic sample size was 249 pregnant women. NHANES protocol was reviewed and approved by the NCHS Research Ethics Review Board [18].

### 2.2. Dietary Assessment

What We Eat in America, component of the NHANES 2003–2012 collected dietary information by using an interviewer-administered 24 h recall that used automated multiple pass methodology developed by the U.S. Department of Agriculture (USDA) [19]. A second dietary recall, 3–10 days after the first dietary recall, was obtained by using phone calls [20]. Although two 24 h dietary recalls were collected in the 2007–2010 NHANES, only the first recall data are recommended to be used by the NCHS as different methods were used to collect dietary data, *i.e.*, day 1 by in-person and day 2 by phone calls [20]. A single 24 h recall has also been reported to be adequate to estimate mean group dietary intake [21].

Dietary pattern analysis was performed in two steps to identify dietary patterns as predictors of the responses to GDM. In the first step, food items were aggregated into 28 food groups, which are comparable with the grouping schemes reported in the Food Patterns Equivalents Database (FPED) 2011–2012 [22] (as shown in Table 1). The USDA’s food code from an individual’s day 1 dietary recall of NHANES was matched to the USDA food code of FPED 2011–2012. Since the components of FPED 2011–2012 are presented per 100 g of food and beverages, an individual’s food intake in grams was divided by 100 g and multiplied by the number of FPED equivalents in FPED 2011–2012 [23]. To derive optimal dietary patterns, total fruit, total vegetables, total red and orange vegetables, total starch vegetables, total grains, total protein foods, total meat, poultry, and seafood, and total dairy from the original FPED 2011–2012’s subgroups were removed because a total subgroup is the summation of its subgroup components. For example, total dairy is the summation of milk, yogurt, and cheese. In the second step, dietary pattern analysis was performed with the reduced rank regression method. The reduced rank regression method extracts linear combinations from predicting variables while maximizing the variance explained within a set of response variables [13]. We used PROC PLS with the reduced rank regression method option to drive dietary patterns using SAS software (version 9.3; SAS Institute, Cary, NC, U.S.). The analysis began with the selection of the 28 food groups on the basis of the number of cup equivalents of fruit, vegetables, and dairy; ounce equivalents of grains and protein foods; teaspoon equivalents of added sugars; gram equivalents of solid fats and oils; and number of alcoholic drinks as independent or exposure variables. This was followed by the choice of the prepregnancy BMI, nutrient intake, and maternal biomarkers related to GDM as response measures following log transformation. The predicting variables are the food groups from a 24 h recall, and the final set of response measures are prepregnancy BMI, dietary fiber, and poly- and monounsaturated fatty acids to saturated fatty acid. The final number of response variables indicating the greatest explanation of the total variation in foods groups and in biomarkers was obtained by sensitivity analysis (Table S1).

**Table 1:** Food patterns equivalents database (FPED) 2011–2012 food groups and modified groups used in the present study.

FPED ^1^ 2011–2012 Food Groups	Original FPED 2011–2012 Subgroups	Modified FPED 2011–2012 Subgroups
Fruit	1. Total fruit	*Removed*
	2. Citrus, melons, and berries	1. Citrus, melons, and berries
	3. Other fruits	2. Other fruits
	4. Fruit juice	3. Fruit juice
Vegetables	5. Total vegetables	*Removed*
	6. Dark green vegetables	4. Dark green vegetables
	7. Total red and orange vegetables	*Removed*
	8. Tomatoes	5. Tomatoes
	9. Other red and orange vegetables (excludes, tomatoes)	6. Other red and orange vegetables (excludes, tomatoes)
	10. Total starchy vegetables	*Removed*
	11. Potatoes (white potatoes)	7. Potatoes (white potatoes)
	12. Other starchy vegetables (excludes white potatoes)	8. Other starchy vegetables (excludes white potatoes)
	13. Other vegetables	9. Other vegetables
	14. Beans and peas computed as vegetables	10. Beans and peas computed as vegetables
Grains	15. Total grains	*Removed*
	16. Whole grains	11. Whole grains
	17. Refined grains	12. Refined grains
Protein Foods	18. Total protein foods	*Removed*
	19. Total meat, poultry, and seafood	*Removed*
	20. Meat (beef, veal, pork, lamb, game)	13. Meat (beef, veal, pork, lamb, game)
	21. Cured meat (frankfurters, sausage, corned beef, cured ham and luncheon meat made from beef, pork, poultry)	14. Cured meat (frankfurters, sausage, corned beef, cured ham and luncheon meat made from beef, pork, poultry)
	22. Organ meat (from beef, veal, pork, lamb, game, poultry)	15. Organ meat (from beef, veal, pork, lamb, game, poultry)
	23. Poultry (chicken, turkey, other fowl)	16. Poultry (chicken, turkey, other fowl)
	24. Seafood high in *n*-3 fatty acids	17. Seafood high in *n*-3 fatty acids
	25. Seafood low in *n*-3 fatty acids	18. Seafood low in *n*-3 fatty acids
	26. Eggs	19. Eggs
	27. Soybean products (excludes calcium fortified soy milk and mature soybeans)	20. Soybean products (excludes calcium fortified soy milk and mature soybeans)
	28. Nuts and seeds	21. Nuts and seeds
	29. Beans and peas computed as protein foods	*Removed*
Dairy	30. Total dairy (milk, yogurt, cheese, whey)	*Removed*
	31. Milk (includes calcium fortified soy milk)	22. Milk (includes calcium fortified soy milk)
	32. Yogurt	23. Yogurt
	33. Cheese	24. Cheese
Oils	34. Oils	25. Oils
Solid Fats	35. Solid fats	26. Solid fats
Added Sugars	36. Added sugars	27. Added sugars
Alcoholic Drinks	37. Alcoholic drinks	28. Alcoholic drinks

USDA’s Food Patterns Equivalents Database 2011–2012 (FPED 2011–2012) converts foods and beverages in the Food and Nutrient Database for Dietary Studies (FNDDS) 2011–2012 to 37 Food Patterns (FP) components [23]. ^1^ The FPED provides an unique research tool to evaluate food and beverage intakes of Americans compared to recommendations of the 2010 Dietary Guidelines for Americans.

The relationship between the 28 food groups and the identified dietary patterns was indicated by factor loadings, which represent the correlation coefficients between the food groups and the dietary patterns. The dietary patterns were labeled on the basis of food groups that loaded highest and/or lowest in the respective dietary pattern. Each pregnant woman was assigned a score of the derived dietary patterns, calculated as the product of the food group value and its factor loading and summed across the food groups.

### 2.3. Maternal Biomarkers

All the blood measurements used in this study were drawn, analyzed, and reported as part of the NHANES 2003–2012 surveys dataset. A fasting blood glucose test was performed on eligible participants who were examined in the morning session after a nine-hour fast [24]. Plasma glucose was measured using an enzyme hexokinase method [24]. For NHANES 2003–2004, glucose and insulin measurements were performed by Diabetes Diagnostic Laboratory at University of Missouri (Columbia, MO, USA) [25], and for NHANES 2005–2012, glucose and insulin measurements were performed by the Fairview Medical Center Laboratory at the University of Minnesota (Minneapolis, MN, USA) [24]. Insulin was measured using Tosoh AIA-PACK IRI immunoenzymometric assay in NHANES 2003–2004 [25], and the Merocodia Insulin ELISA Immunoassay in NHANES 2005–2012 [24]. Insulin resistance was estimated using the homeostatic model assessment for insulin resistance (HOMA-IR) by the following formula: fasting insulin (μU/mL) × fasting glucose (mmol/L)/22.5 [26]. Glycohemoglobin (HbA1C) was measured using a Tosoh A1C 2.2 Plus Glycohemoglobin Analyzer (Tosoh Medics Inc., San Francisco, CA, USA) or a Tosoh G7 Automated HPLC Analyzer (Tosoh Medics Inc., San Francisco, CA, USA) [27]. CRP (nmol/L) was measured by latex-enhanced nephelometry [28]. Vitamin C (µmol/L) level in serum was measured using isocratic high performance liquid chromatography (HPLC) with electrochemical detection at 650 mV1 [29]. Lastly, vitamin D (nmol/L) concentration was measured by using the Diasorin 25-OH-Vitamin D assay (DiaSorin Inc., Stillwater, MN, USA) [30].

### 2.4. Outcome Variables

In this cross-sectional study, the average gestational age of study participants was 20 weeks. GDM was diagnosed according to the 2010 International Association of Diabetes and Pregnancy Study Groups (IADPSG) Consensus Panel [31] if the following criteria were met: fasting plasma glucose level ≥ 5.1 mmol/L before 24 weeks of gestation.

### 2.5. Covariates

Analyses were adjusted for maternal age, race/ethnicity, family poverty income ratio, education, marital status, and physical activity level. Maternal age was controlled in continuous variables. The study group consisted of Mexican-American or other Hispanic, non-Hispanic White, non-Hispanic Black and other race. Family poverty income ratio was divided into three categories: ≤1.85, 1.85–4 and >4. Maternal education was grouped by the number of completed years of school: less than high school, high school diploma and more than high school. Marital status was divided into three groups: married/living with a partner, widowed/divorced/separated and single. Physical activity level was divided into four groups: no activity, 0–500 MET-minutes/week, 500–1000 MET-minutes/week and ≥1000 MET minutes/week.

### 2.6. Statistical Analyses

Maternal characteristics were expressed as numbers (weighted percentages) by the status of GDM. The Chi-square test was performed to test the association between maternal characteristic and the status of GDM. The risk for GDM was categorized as yes or no, and multivariable logistic regression models were applied to estimate odds ratios (ORs) (95% CI) of the risk for GDM across tertiles of dietary pattern scores. The *p* for trend across tertiles was computed by treating dietary pattern scores as continuous variables. We first ran models testing crude associations, then models were adjusted in three ways: (1) maternal age, race/ethnicity, education, family poverty income ratio and marital status; (2) model 1 + prepregnancy BMI + gestational weight gain + energy intake; (3) model 2 + log-transformed CRP concentrations.

To analyze the magnitude of collinearity, the variance inflation factor (VIF) was used to test with VIF <5 set as the acceptable level [32]. NHANES uses a complex sample survey design including a multistage cluster sample and weighting methodology that oversamples certain groups of individuals to ensure adequate statistical power. All analyses were carried out using SAS software, which incorporates appropriate sampling weights to adjust for the complex sampling weights. Sampling weights associated with the smallest subsample (fasting subsample) were used as recommended by the NHANES [33].

## 3. Results

Pregnant women’s characteristics according to the status of GDM are shown in Table 2. Pregnant women with GDM generally had a family poverty income ratio ≤1.85 and were less likely to be involved in physical activity compared to women without GDM. Multi-collinearity between age, race/ethnicity, family poverty income ratio, education, marital status, and physical activity did not exist. The VIF for the all confounding variables were less than 2.

**Table 2:** Maternal characteristics in relation to gestational diabetes mellitus (GDM).

	GDM		No GDM		*p* Value ^2^
	*n*	Wt’d% ^1^	*n*	Wt’d% ^1^	
**Age**					
≤25	17	57.4	94	37.2	0.22
26–35	14	38.7	110	56.0	
≥35	3	3.9	11	6.8	
**Race**					
Mexican American or other Hispanic	10	20.9	74	21.3	0.60
Non-Hispanic white	18	62.9	96	54.7	
Non-Hispanic black	4	14.3	32	14.3	
Other including multi-racial	2	1.9	13	9.6	
**Family poverty income ratio**					
≤1.85	20	62.6	108	38.1	0.02
1.85–4	5	5.9	60	35.8	
>4	9	31.5	47	26.1	
**Education level**					
≤11th Grade	12	37.7	75	21.2	0.23
High School Grade	5	8.2	38	19.7	
Above College	17	54.1	102	59.1	
**Marital status**					
Married or living with a partner	29	86.8	171	85.9	0.76
Widowed/divorced/separated	1	4.1	7	2.2	
Single	4	9.2	37	11.9	
**Parity (*n =* 179)**					
None	1	12.7	12	6.7	0.46
1	14	51.7	79	47.2	
2	10	34.6	38	33.0	
≥3	1	1.1	24	13.0	
**Trimester of pregnancy**					
1st trimester	12	50.9	39	29.3	0.15
2nd trimester	12	26.9	86	34.2	
3rd trimester	10	22.3	90	36.5	
**Prepregnancy weight status**					
BMI < 25 kg/m^2^	8	29.4	133	61.7	0.06
BMI ≥ 25 kg/m^2^	26	70.6	82	38.2	
**Gestational weight gain**					
Inadequate	8	14.9	59	29.9	0.07
Adequate	3	12.0	49	23.4	
Excessive	23	73.0	107	46.7	
**Physical activity (*n =* 154)**					
None	6	29.1	8	10.1	0.02
0 to <500 MET-min/week	10	44.7	66	48.8	
500 to <1,000 MET-min/week	5	21.8	23	14.9	
≥1000 MET-min/week	3	4.4	33	26.3	
**C-reactive protein**					
>28.6 nmol/L	29	82.0	164	73.5	0.41
≤28.6 nmol/L	5	18.0	51	26.5	

^1^ Wt’d%: Weighted %. Sample weights are created in the National Health and Nutrition Examination Survey (NHANES) to account for the complex survey design (including oversampling of some subgroups), survey non-responses, and post-stratification. When a sample is weighted in NHANES, it is representative of the U.S. civilian non-institutionalized Census population. Weighted percentages may not sum up to 100 due to rounding. ^2^ *p* Value obtained from Chi-square tests.

Dietary patterns were derived using the reduced rank regression method. The reduced rank regression method derives dietary patterns from predictors to maximize the explained variation of pre-defined set of responses chosen [34]. Responses chosen for reduced rank regression were prepregnancy BMI and nutrients that have bene consistently associated with GDM in the literature such as dietary fiber and ratio of poly- and monounsaturated fatty acids to saturated fatty acids [4,5,35]. Sensitivity analysis using different numbers of response variables (different sets for prepregnancy BMI and GDM-related nutrients including or excluding GDM-related biomarkers) indicated that the greatest explanation of the total variation in foods and in responses was obtained using prepregnancy BMI, dietary fiber, and ratio of poly- and monounsaturated fatty acids to saturated fatty acids (Table S1). Three factors were extracted with reduced rank regression, explaining the 45.9% of the total variation in the response variables and the 15.0% variation in food groups (Table S2). Three dietary patterns were derived using reduced rank regression. Loading values for each of the 28 food groups for the reduced rank regression obtained dietary patterns are presented in Table S3. The “high refined grains, fats, oils and fruit juice” pattern was characterized by high loadings of refined grains, solid fats, oils, and fruit juice. The “high nuts, seeds, fat and soybean; low milk and cheese” pattern was characterized by high loadings of nuts and seeds, solid fats, soybean products and low loadings of milk and cheese. The “high added sugar and organ meats; low fruits, vegetables and seafood” pattern was represented by high loadings of added sugars and organ meats and low loadings of fruits and vegetables and seafood (Table S3).

Maternal characteristics according to the tertiles of three dietary patterns’ scores are presented in Table 3. Total energy intake and dietary fiber intake were differed significantly by the tertiles of “high refined grains, fats, oils and fruit juice” dietary pattern score. Total energy intake, total fat and saturated fat intake as percentages of energy, dietary fiber, ratio of poly- and monounsaturated fatty acids to saturated fatty acid, and serum vitamin D significantly differed by the tertiles of “high nuts, seeds, fat and soybean; low milk and cheese” dietary pattern score. Prepregnancy BMI, carbohydrate, protein and monounsaturated fatty acids intake as percentages of energy, dietary fiber, and HOMA-IR significantly differed by the tertiles of “high added sugar and organ meats; low fruits, vegetables and seafood” dietary pattern score (Table 3).

**Table 3:** Maternal characteristics by the tertiles of dietary pattern scores.

	“High Refined Grains, Fats, Oils and Fruit Juice” Pattern				“High Nuts, Seeds, Fat and Soybean; Low Milk and Cheese” Pattern				“High Added Sugar and Organ Meats; Low Fruits, Vegetables and Seafood” Pattern			
	Tertile 1 (*n =* 83)	Tertile 2 (*n =* 83)	Tertile 3 (*n =* 83)	*p* trend	Tertile 1 (*n =* 83)	Tertile 2 (*n =* 83)	Tertile 3 (*n =* 83)	*p* trend	Tertile 1 (*n =* 83)	Tertile 2 (*n =* 83)	Tertile 3 (*n =* 83)	*p* trend
Age (year)	25.7 ± 0.6 ^1^	28 ± 0.9	27.5 ± 0.7	0.06	28.6 ± 0.8	26.8 ± 079	26.0 ± 0.6	0.05	28.8 ± 0.9	26.6 ± 0.6	26.6 ± 0.8	0.09
Prepregnancy BMI (kg/m^2^)	25.4 ± 1.2	27.6 ± 0.9	25.4 ± 0.7	0.07	26.0 ± 1.3	25.8 ± 0.9	26.8 ± 1.1	0.76	24.9 ± 1.2	24.6 ± 0.8	28.3 ± 1.1	0.008
Total energy (kcal/day)	1985.0 ± 125.1	2539.2 ± 137.7	2811.3 ± 153.7	0.0007	2866.5 ± 113.0	2116.6 ± 118.0	2320.3 ± 105.5	<0.0001	2658.1 ± 127.1	2230.0 ± 187.5	2463.5 ± 96.1	0.12
Carbohydrate (% of energy/day)	53.1 ± 2.3	52.7 ± 1.1	54.1 ± 1.7	0.82	52.7 ± 1.9	55.7 ± 1.5	50.9 ± 1.4	0.05	48.9 ± 1.4	51.6 ± 1.2	57.3 ± 1.7	0.008
Protein (% of energy/day)	15.9 ± 0.8	14.0 ± 0.4	14.1 ± 0.5	0.09	14.7 ± 0.6	14.9 ± 0.7	14.4 ± 0.5	0.83	16.6 ± 1.0	15.5 ± 0.9	12.8 ± 0.5	<0.0001
Total fat (% of energy/day)	31.7 ± 1.9	34.5 ± 1.2	33.9 ± 1.3	0.46	33.7 ± 1.6	30.8 ± 1.3	36.1 ± 1.1	0.02	35.6 ± 1.1	33.8 ± 1.6	31.6 ± 1.3	0.05
MUFA (% of energy/day)	11.9 ± 0.8	12.6 ± 0.5	12.2 ± 0.5	0.73	12.2 ± 0.7	11.6 ± 0.5	13.1 ± 0.5	0.12	13.0 ± 0.4	12.7 ± 0.6	11.4 ± 0.5	0.02
SFA (% of energy/day)	10.8 ± 0.7	11.5 ± 0.6	11.0 ± 0.6	0.69	12.4 ± 0.6	10.5 ± 0.7	10.5 ± 0.5	0.04	12.2 ± 0.6	10.9 ± 0.8	10.6 ± 0.5	0.15
Dietary fiber (g/day)	10.9 ± 1.0	16.6 ± 0.6	26.1 ± 1.2	<0.0001	20.7 ± 1.2	15.8 ± 1.5	15.7 ± 0.8	0.004	19.9 ± 1.3	14.8 ± 1.3	18.3 ± 1.3	0.03
Fatty acids ratio ^2^	1.6 ± 0.1	1.6 ± 0.1	1.8 ± 0.1	0.54	1.3 ± 0.1	1.6 ± 0.1	2.1 ± 0.1	<0.0001	1.6 ± 0.1	1.8 ± 0.1	1.6 ± 0.1	0.59
Glycohemoglobin (%)	4.9 ± 0.1	4.9 ± 0.1	5.0 ± 0.1	0.34	4.9 ± 0.1	5.0 ± 0.1	5.0 ± 0.1	0.54	5.0 ± 0.1	4.9 ± 0.1	5.0 ± 0.1	0.50
HOMA-IR	2.2 ± 0.2	2.5 ± 0.3	2.4 ± 0.3	0.67	2.2 ± 0.2	2.1 ± 0.2	2.9 ± 0.4	0.25	2.2 ± 0.2	1.8 ± 0.2	3.0 ± 0.3	0.02
Fasting glucose (mmol/L)	4.7 ± 0.1	4.6 ± 0.1	4.8 ± 0.1	0.20	4.6 ± 0.1	4.8 ± 0.1	4.7 ± 0.1	0.36	4.6 ± 0.1	4.6 ± 0.1	4.8 ± 0.1	0.08
Serum Vitamin C (µmol/L)	62.5 ± 4.5	56.8 ± 3.4	68.1 ± 3.4	0.15	62.5 ± 2.8	68.1 ± 4.0	62.5 ± 5.1	0.78	62.5 ± 2.3	73.8 ± 3.4	56.8 ± 5.1	0.12
Serum Vitamin D (nmol/L)	65.4 ± 4.7	77.6 ± 8.0	70.6 ± 6.0	0.36	72.6 ± 4.0	78.1 ± 8.2	61.2 ± 4.2	0.04	70.1 ± 3.7	77.1 ± 8.7	67.4 ± 5.5	0.59
C-reactive protein (nmol/L)	57.1 ± 6.7	66.7 ± 8.6	57.1 ± 7.6	0.91	47.6 ± 4.8	57.1 ± 5.7	76.2 ± 9.5	0.07	66.7 ± 8.6	57.1 ± 5.7	66.7 ± 6.7	0.24

^1^ Mean ± SE (all such values); ^2^ Ratio of poly- and monounsaturated fatty acids to saturated fatty acid. BMI: body mass index. HOMA-IR: the homeostatic model assessment for insulin resistance. MUFA: monounsaturated fatty acids. SFA: saturated fatty acids.

Covariate-adjusted multivariable logistic regression analyses showed that all three dietary patterns were significantly and positively associated with a higher GDM risk (Table 4). In the fully adjusted multivariable model 4, comparing pregnant women in the highest tertile with those in the lowest reference tertile of “high refined grains, fats, oils and fruit juice” pattern, pregnant women had a higher odds of developing GDM (OR 4.9; 95% CI 1.4–17.0). Pregnant women in the highest tertile of the “high nuts, seeds, fat and soybean; low milk and cheese” pattern had higher odds of GDM (OR 7.5; 95% CI 1.8–32.3) than those in the lowest tertile (model 4). Pregnant women in the highest tertile of “high added sugar and organ meats; low fruits, vegetables and seafood” pattern had higher odds of GDM (OR 21.1; 95% CI 4.0–109.8) than those in the lowest tertile (model 3). The significant relationship between the “added sugar, low fruits and vegetables” diet and GDM persisted after controlling for log-transformed CRP (OR 22.3; 95% CI 3.9–127.4) (model 4).

**Table 4:** Odds ratios (and 95% CIs) for risk of gestational diabetes mellitus (GDM) according to the tertiles of dietary pattern score derived from reduced rank regression (*n =* 249).

	Tertile 1	Tertile 2	Tertile 3	*p* Trend
“High Refined Grains, Fats, Oils and Fruit Juice” Pattern				
GDM/pregnancies	8/83	11/83	15/83	
Model 1	1.0	1.1 (0.3–3.9)	3.7 (0.9–15.7)	0.09
Model 2	1.0	1.7 (0.5–5.8)	5.1 (1.1–24.0) *	0.04
Model 3	1.0	1.3 (0.5–3.7)	4.9 (1.4–17.3) *	0.009
Model 4	1.0	1.4 (0.4–4.5)	4.9 (1.4–17.0) *	0.007
“High Nuts, Seeds, Fat and Soybean; Low Milk and Cheese” Pattern				
GDM/pregnancies	9/83	11/83	14/83	
Model 1	1.0	4.7 (1.9–11.5) *	5.2 (2.2–12.2) *	0.004
Model 2	1.0	4.2 (1.6–11.1) *	5.7 (2.1–15.2) *	0.001
Model 3	1.0	5.5 (2.5–12.1) *	8.2 (1.8–37.4) *	0.01
Model 4	1.0	5.3 (2.3–12.2) *	7.5 (1.8–32.3) *	0.009
“High Added Sugar and Organ Meats; Low Fruits, Vegetables and Seafood” Pattern				
GDM/pregnancies	5/83	8/83	21/83	
Model 1	1.0	1.7 (0.4–7.0)	15.4 (4.5–52.0) *	0.0004
Model 2	1.0	2.2 (0.3–14.1)	20.0 (4.2–95.9) *	0.0004
Model 3	1.0	2.9 (0.6–13.1)	21.1 (4.0–109.8) *	<0.0001
Model 4	1.0	3.2 (0.7–15.7)	22.3 (3.9–127.4) *	<0.0001

Model 1: Crude association between dietary patterns and gestational diabetes mellitus; Model 2: Adjusted for age, race/ethnicity, family poverty income ratio, education level, and marital status. Model 3: Adjusted for model 2 + energy intake, prepregnancy body mass index (BMI), and gestational weight gain. Model 4: Adjusted for model 3 + log-transformed C-reactive protein (CRP); * *p* < 0.05.

## 4. Discussion

In this cross-sectional study, three dietary patterns during pregnancy were identified with the choice of response variables including prepregnancy BMI, ratio of poly- and monounsaturated fatty acids to saturated fatty acids and dietary fiber: “high refined grains, fats, oils and fruit juice” pattern, “high nuts, seeds, fat and soybean; low milk and cheese” pattern, and “high added sugar and organ meats; low fruits, vegetables and seafood” pattern. Despite small differences, all three dietary patterns were associated with increased risks for GDM. Among three dietary patterns, the strongest relationship to the GDM risk was found for “high added sugar and organ meats; low fruits, vegetables and seafood” pattern. The positive association of the “high added sugar and organ meats; low fruits, vegetables and seafood” pattern with GDM, was largely explained by the high consumption of added sugars and low consumption of fruits and vegetables. Sugar-sweetened beverages are one of the leading sources of added sugars in the American diet [36]. In the Nurses’ Health Study II, intake of sugar-sweetened coke before pregnancy was positively associated with the risk of GDM [37]. Compared to women who consumed one serving/month, those women who consumed ≥5 servings/week of sugar sweetened coke had a 22% greater risk for GDM (relative risk (RR) 1.22; 95% CI 1.01–1.47). Epidemiologic studies demonstrate that high consumption of sugar-sweetened beverages was associated with increased risk for type 2 diabetes among general adult populations [38,39,40]. High sugar intake is also associated with high energy intake and hence obesity which is associated with risk of GDM. The high levels of rapidly absorbable carbohydrates in the form of added sugars of sugar sweetened beverages [40] may increase the levels of fasting blood glucose levels and insulin resistance. In our study, low intake of fruits and vegetables pattern was associated with an increased risk for GDM. Although the biological mechanisms for the inverse associations of fruits and vegetable intake and GDM risk are not clear, Bazzano *et al.* [41] explained that fruit and green leafy vegetables may contribute to a decreased incidence of type 2 diabetes through their low energy density, low glycemic load and high fiber. This mechanism may partially explain the association of low intake of fruits and vegetables in relation to decreased risk for GDM. Our findings are further supported by the findings from the Nurses’ Health Study II [8]. Women in the lowest quintile of the prudent pattern characterized by a high intake of fruit, vegetables, and green leafy vegetables (lowest adherence) were associated with increased risks for GDM compared to those women in the highest quintile (highest adherence) (RR 1.39; 95% CI 1.08–1.80). In the same prospective cohort of Nurses’ Health Study II, intake of whole fruits and green leafy green vegetables was inversely associated with incidence of type 2 diabetes in the middle-aged U.S. women [42].

The association with “high refined grains, fats, oils and fruit juice” pattern was largely explained by high intakes of refined grains and solid fats. Our findings are in accordance with the evidence of positive associations of the “Western” dietary pattern, characterized by high intakes of refined grains and solid fats with GDM in pregnant women [8]. In the Nurses’ Health Study II [8], the “Western” dietary pattern before pregnancy characterized by high intake of red meat, processed meat, refined grain products, and sweets were associated with the risk of GDM. In contrast, the “Western” dietary pattern in the first month of pregnancy, which included red and processed meats, sugar-sweetened beverages, and refined grains, was not associated with the risk of GDM in the prospective cohort study of Project Viva [43]. The authors explained that once insulin resistance has been established from years of dietary patterns characterized by the “Western” dietary pattern, what women eat in the first few months of pregnancy may not have additional effect on the risk of GDM.

The positive association of “high nuts, seeds, fat and soybean; low milk and cheese” pattern with GDM was partly explained by low intakes of fruits, tomatoes, and beans and peas although high nuts and seeds would be expected to be protective on GDM risk. Low intake of fruits may partially explain the positive association between “high nuts, seeds, fat and soybean; low milk and cheese” pattern and the risk for GDM. Low consumption of fruits, lack of phytonutrients, including carotenoids and vitamins such as vitamin C [44], found to have preventive effect on GDM [45] may explain the association.

There are inconsistent findings regarding the relationship between elevated CRP and the risk for GDM. Elevated maternal CRP concentration in the first trimester of pregnancy has been reported to be positively associated with the risk for GDM in the third trimester [46,47]. In contrast, maternal serum levels of CRP were not associated with the risk for GDM but significantly correlated with prepregnancy obesity in a cross-sectional study [48]. In our study, CRP levels (≤28.6 nmol/L *vs.* >28.6 nmol/L) were not significantly differed by the status for GDM. For this reason, after adjustment for CRP levels, the significant relationship between dietary patterns and the risk for GDM persisted.

The strengths of this study are that first, the reduced rank regression method allowed for a hypothesis regarding pathways (by the response variables) between diet and disease (GDM) to be evaluated [34]. Although traditional principal component analysis seems beneficial in the past, the pattern solely focused on inter-correlations among food groups, which may not represent diet qualities relevant to specific disease etiology [34]. Reduced rank regression is useful for etiological investigation explaining how a certain dietary pattern is associated with the health outcome of interest [49]. In our study, a great number of potential confounders such as physical activity, prepregnancy BMI and gestational weight gain were controlled in the analysis. Lastly, we also demonstrated that multi-collinearity among covariates did not exist.

The study has several limitations. Due to the use of cross-sectional study design of NHANES, we cannot provide evidence of a causal relationship between dietary patterns during pregnancy and the risk for GDM. Particularly, this could be the result of reverse causality in which subjects may change or adapt to different styles of diet after the diagnosis for GDM. Another limitation is that a history of family type 2 diabetes was not controlled for in our analysis. Due to the relatively small sample size of pregnant women included in this study, low statistical power may cause the wide confidence intervals in our analysis. It is possible that women with GDM are consuming foods high in added sugars and solid fats without recognizing that they are diagnosed with GDM. Lastly, FFQ would have been better to capture dietary patterns than 24 h recalls.

## 5. Conclusions

In conclusion, dietary patterns during pregnancy were associated with increased risks for GDM. Women in the third tertile of “high refined grains, fats, oils and fruit juice”, “high nuts, seeds, fat and soybean; low milk and cheese” and “high added sugar and organ meats; low fruits, vegetables and seafood” dietary patterns were all significantly associated with increased risk for GDM. Prospective and cohort studies are needed to further evaluate and monitor changes in dietary patterns before to during pregnancy and its effect on the risk for GDM in consideration of GDM-related lifestyle factors such as physical activity levels.

## Supplementary Materials

The following are available online at www.mdpi.com/2072-6643/7/11/5472/s1, Table S1: Response variables to derive dietary patterns using reduced rank regression, Table S2: Variations explained by food groups and response variables by extracted dietary patterns, Table S3: Loadings of food groups in dietary pattern scores in pregnant women.
