# Supplementary material for: Dietary Patterns during Pregnancy Are Associated with Risk of Gestational Diabetes Mellitus
Source: Nutrients. 2015 Nov 12;7(11):9369–82. doi: 10.3390/nu7115472 (PMC4663600; doi:10.3390/nu7115472)
Supplement: Supplementary file 1 [file nutrients-07-05472-s001.docx]

Supplementary Materials: Dietary Patterns during Pregnancy Are Associated with Risk of Gestational Diabetes Mellitus

Dayeon Shin, Kyung Won Lee and Won O. Song *

**Table S1.** Response variables to derive dietary patterns using reduced rank regression.

| 1^st^ Set | 2^nd^ Set | 3^rd^ Set |
| --- | --- | --- |
| Prepregnancy BMI | Prepregnancy BMI | Prepregnancy BMI |
| Fatty acids ^1^  Dietary fiber | Fatty acids  Dietary fiber | Fatty acids  Dietary fiber |
| Glycohemoglobin | CRP |  |
| HOMA-IR | HOMA-IR |  |
| Glucose | Glucose |  |
| Vitamin C |  |  |
| Vitamin D |  |  |
| CRP |  |  |
| Food Groups: 15.6% ^2^ | Food Groups: 15.0% | Food Groups: 15.0% |
| Responses: 18.6% | Responses: 25.4% | Responses: 45.9% |

^1^ Ratio of poly- and monounsaturated fatty acids to saturated fatty acids; ^2^ Explained variation. BMI: body mass index. HOMA-IR: the homeostatic model assessment for insulin resistance. CRP: C-reactive protein.

**Table S2.** Variations explained by food groups and response variables by extracted dietary patterns.

|  | “**Refined Grains and Solid Fats**” **Dietary Pattern** | “**Nuts, Seeds and Oils**” **Dietary Pattern** | “**Added Sugars, Low Fruits and Vegetables**” **Dietary Pattern** | **Total Explained Variation** |
| --- | --- | --- | --- | --- |
| Food groups | 6.7% | 4.8% | 3.5% | 15.0% |
| Responses | 28.5% | 14.9% | 2.5% | 45.9% |

Dietary patterns obtained with reduced rank regression using pregnancy body mass index (BMI), ratio of poly- and monounsaturated fatty acids to saturated fatty acids, and dietary fiber as response variables in the procedure.

**Table S3.** Loadings of food groups in dietary pattern scores in pregnant women.

| **No.** | **Food Group** | “**High Refined Grains, Fats, Oils and Fruit Juice**” **Pattern** | “**High Nuts, Seeds, Fat and Soybean; Low Milk and Cheese**” **Pattern** | “**High Added Sugar and Organ Meats; Low Fruits, Vegetables and Seafood**” **Pattern** |
| --- | --- | --- | --- | --- |
| 1 | Other starchy vegetables (excludes white potatoes) | **0.14** | −0.03 | −0.01 |
| 2 | Refined grains | **0.30** | −**0.14** | 0.03 |
| 3 | Whole grains | **0.26** | −0.01 | 0.03 |
| 4 | Milk (includes calcium fortified soy milk) | **0.12** | −**0.33** | −0.02 |
| 5 | Other fruits | **0.26** | −**0.21** | −0.06 |
| 6 | Tomatoes | **0.26** | −**0.13** | −0.01 |
| 7 | Soybean products (excludes calcium fortified soy milk and mature soybeans) | **0.17** | **0.41** | −**0.24** |
| 8 | Other vegetables | **0.24** | **0.17** | −**0.28** |
| 9 | Beans and peas | **0.36** | −**0.19** | **0.25** |
| 10 | Nuts and seeds | **0.26** | **0.24** | **0.17** |
| 11 | Citrus, melons, and berries | **0.20** | −**0.13** | −**0.21** |
| 12 | Cheese | **0.17** | −**0.25** | −**0.10** |
| 13 | Oils | **0.23** | **0.44** | **0.15** |
| 14 | Solid fats | **0.14** | −**0.40** | −**0.19** |
| 15 | Fruit juice | **0.14** | 0.08 | −**0.17** |
| 16 | Other red and orange vegetables (excludes, tomatoes) | **0.23** | 0.05 | −**0.17** |
| 17 | Potatoes (white potatoes) | **0.17** | 0.09 | −**0.25** |
| 18 | Meat (beef, veal, pork, lamb, game) | 0.03 | −**0.15** | −**0.38** |
| 19 | Dark green vegetables | 0.09 | 0.00 | **0.15** |
| 20 | Added sugars | −0.04 | 0.01 | **0.28** |
| 21 | Alcoholic drinks | −0.03 | 0.01 | −**0.12** |
| 22 | Organ meat (from beef, veal, pork, lamb, game, poultry) | 0.03 | −0.01 | **0.12** |
| 23 | Cured meat (frankfurters, sausage, corned beef, cured ham and luncheon meat made from beef, pork, poultry) | −0.06 | −0.02 | −**0.11** |
| 24 | Seafood low in n-3 fatty acids | −0.01 | 0.05 | −**0.11** |
| 25 | Eggs | 0.03 | −0.05 | −**0.40** |
| 26 | Poultry (chicken, turkey, other fowl) | 0.04 | 0.09 | −0.03 |
| 27 | Seafood high in n-3 fatty acids | 0.03 | 0.06 | 0.02 |
| 28 | Yogurt | 0.06 | −0.06 | −0.06 |

Factor loadings represent the magnitude and direction of association with factors (dietary patterns) and can range from −1.0 to 1.0. Food groups with factor loading values ≥ |0.10| are indicated in bold. No.: number.
